# Behavioral Health, Social Engagement, and Long-Term Care Services Use Among Community Older Adults: USA vs. Taiwan

**DOI:** 10.1093/geroni/igab046.1344

**Published:** 2021-12-17

**Authors:** Su-I Hou, Chien-Ching Li, Darren Liu

## Abstract

As healthcare advances, older adults are living longer. While 90% of older adults prefer aging in their own homes and communities, it is important to examine key factors influencing healthy aging-in-community and community-based long-term care (LTC) services available in different countries. This symposium examines behavioral health, social engagement, and LTC services utilization among community-dwelling older adults in the USA and Taiwan. Lessons learned from older adults across countries will provide insights for tailored community-based LTC services and program development. Dr. Hou from The University of Central Florida (UCF) will highlight similarities and differences in behavioral health profiles and the topics that most interest community-dwelling older Americans participating in three aging-in-community programs in Central Florida. Dr. Wang from Case Western Reserve University will examine the impact of neighborhood social cohesion on mobility among community-dwelling older Americans aged 65 and older from the national Health and Retirement Study. Dr. Liu from National Cheng-Kung University in Taiwan will share results of healthy lifestyle on quality of life among community-dwelling older adults in southern Taiwan. Dr. Young from State University of New York at Albany will compare long-term care use among community-dwelling older adults with and without dementia in Central Taiwan. Finally, Drs. Cao and Hou from UCF will analyze home and community-based services in the USA versus Taiwan. This symposium will further discuss similarities and differences of key factors related to healthy aging-in-community, along with practical recommendations and lessons learned across countries and cultural environments to improve community-based long-term care services and programs.

